# The Paradox of Digital Ageism: Cultural and Age Differences in Technology Use Between China and the U.S.

**DOI:** 10.1093/geroni/igaf122.918

**Published:** 2025-12-31

**Authors:** Wanyu Xi, Ittay Mannheim, Yuxiang (Chris) Zhao, Ella Cohn-Schwartz, Yaacov G Bachner

**Affiliations:** Nanjing University of Chinese Medicine, Nanjing, Jiangsu, China (People’s Republic); Ben Gurion University of the Negev, Be’er Sheva, HaDarom, Israel; Nanjing University, Nanjing, Jiangsu, China (People’s Republic); Ben Gurion University of the Negev, Be’er Sheva, HaDarom, Israel; Ben Gurion University of the Negev, Be’er Sheva, HaDarom, Israel

## Abstract

Digital ageism—the stereotyping, prejudice, and discrimination against older adults in digital contexts—is an increasing challenge alongside global digitalization. As ageism differs between Eastern and Western cultures and digital progress varies worldwide, this study reveals a paradox in digital ageism between China and the US, examining its impact on technology use. In 2024, we surveyed 410 Chinese participants (ages 18-86, Mage=51.49, SD = 17.86, 65.9% women) and 398 Americans (ages 18-85, Mage=51.63, SD = 17.00, 48.5% women) using both online and paper questionnaires. Measurements included digital ageism, measured with the Attitudes Towards Older Adults Using Technology (ATOAUT) scale, daily technology use, and demographic covariates. A two-way ANOVA revealed significantly higher levels of digital ageism among Chinese participants overall. Paradoxically, older Chinese exhibited higher digital ageism than younger Chinese, while older Americans displayed less ageism than younger Americans. Moreover, a regression analysis showed that, after controlling for demographic factors, higher digital ageism may suppress daily technology engagement for middle-aged and older Chinese (rather than younger adults), yet it unexpectedly boosted younger and middle-aged Americans’ technology use (rather than older adults). These findings suggest that middle-aged and older adults in China, which is undergoing rapid digitalization, are more likely to internalize digital ageism, despite filial piety valuing elder respect. In contrast, older adults in America, where individualism is emphasized, may defy self-ageism, thus avoiding following negative impact. By illuminating digital ageism’s cultural and age-specific dynamics, this research highlights the need for targeted interventions to promote inclusive technology use across diverse societies.

